# *Hyssopus* Essential Oil: An Update of Its Phytochemistry, Biological Activities, and Safety Profile

**DOI:** 10.1155/2022/8442734

**Published:** 2022-01-13

**Authors:** Javad Sharifi-Rad, Cristina Quispe, Manoj Kumar, Muhammad Akram, Mewish Amin, Mehwish Iqbal, Niranjan Koirala, Oksana Sytar, Dorota Kregiel, Silvana Nicola, Andrea Ertani, Montserrat Victoriano, Nafiseh Khosravi-Dehaghi, Miquel Martorell, Mohammed M. Alshehri, Monica Butnariu, Marius Pentea, Lia Sanda Rotariu, Daniela Calina, Natália Cruz-Martins, William C. Cho

**Affiliations:** ^1^Facultad de Medicina, Universidad del Azuay, Cuenca, Ecuador; ^2^Facultad de Ciencias de la Salud, Universidad Arturo Prat, Avda. Arturo Prat 2120, Iquique 1110939, Chile; ^3^Chemical and Biochemical Processing Division, ICAR-Central Institute for Research on Cotton Technology, 400019, Mumbai, India; ^4^Department of Eastern Medicine, Government College University Faisalabad, Pakistan; ^5^Institute of Health Management, Dow University of Health Sciences, Karachi, Pakistan; ^6^Department of Natural Products Research, Dr. Koirala Research Institute for Biotechnology and Biodiversity, Kathmandu 44600, Nepal; ^7^Faculty of Science and Technology, University of Macau, Macau SAR 999078, China; ^8^Department of Plant Biology Department, Taras Shevchenko National University of Kyiv, Institute of Biology, Volodymyrska str., 64, Kyiv 01033, Ukraine; ^9^Department of Plant Physiology, Slovak University of Agriculture, Nitra A. Hlinku 2, 94976 Nitra, Slovakia; ^10^Technical University of Lodz, Faculty of Biotechnology and Food Sciences, 90-924 Lodz, Poland Wolczanska 171/173; ^11^Department of Agricultural, Forest and Food Sciences, University of Turin, Italy; ^12^Department of Nutrition and Dietetics, Faculty of Pharmacy, University of Concepcion, Concepcion 4070386, Chile; ^13^Evidence-Based Phytotherapy & Complementary Medicine Research Center, Alborz University of Medical Sciences, Karaj, Iran; ^14^Department of Pharmacognosy, School of Pharmacy, Alborz University of Medical Sciences, Karaj, Iran; ^15^Centre for Healthy Living, University of Concepción, Concepción 4070386, Chile; ^16^Department of Pharmaceutical Care, Ministry of National Guard-Health Affairs, Riyadh, Saudi Arabia; ^17^Banat's University of Agricultural Sciences and Veterinary Medicine “King Michael I of Romania” From Timisoara, 300645, Calea 119, Timis, Romania; ^18^Department of Clinical Pharmacy, University of Medicine and Pharmacy of Craiova, 200349 Craiova, Romania; ^19^Faculty of Medicine, University of Porto, Alameda Prof. Hernani Monteiro, 4200-319 Porto, Portugal; ^20^Institute for Research and Innovation in Health (i3S), University of Porto, 4200-135 Porto, Portugal; ^21^Institute of Research and Advanced Training in Health Sciences and Technologies (CESPU), Rua Central de Gandra, 1317, 4585-116 Gandra PRD, Portugal; ^22^TOXRUN-Toxicology Research Unit, University Institute of Health Sciences, CESPU, CRL, 4585-116 Gandra, Portugal; ^23^Department of Clinical Oncology, Queen Elizabeth Hospital, Kowloon, Hong Kong

## Abstract

The genus *Hyssopus* is widespread in central Asia, East Mediterranean, and Mongolian areas. It has six main species which are used as herbal remedies, such as *Hyssopus officinalis* which is used as a condiment and flavoring agent in food industry. The other five species are *H. ambiguus*, *H. cuspidatus*, *H. latilabiatus*, *H. macranthus*, and *H. seravschanicus*. Its species are used in the treatment of various ailments such as cold, cough, loss of appetite, fungal infection, and spasmodic condition. Its constituents especially essential oils are popularly used as an additive in beverages, foods, and cosmetics. The volatile constituents are used for aroma in the food industry, cosmetic industry, and household products. The important active constituents in its essential oils are *β*-pinene, pinocamphone, isopinocamphone, and other terpenoids. *Hyssopus* genus is also bundled with other secondary metabolites including flavonoids luteolin, quercetin, apigenin, and their glucosides, as well as phenolic compounds including ferulic, *p*-hydroxy-benzoic acid, protocatechuic acid, chlorogenic, and caffeic acid. Combinedly, the extracts of *Hyssopus* are reported to have potential antiviral and antifungal activities proven using *in vitro* studies, whereas in vivo investigations have reported the crucial role of Hyssopus extracts in plasma membrane relaxation, cytotoxic, and sedative effects. This plant is believed to be relatively safe at levels commonly used in foods; nevertheless, more studies are needed to determine the safety profile.

## 1. Introduction

*Hyssopus* (Lamiaceae) is a genus of herbaceous or semiwoody plants that includes about 10 to 15 species, distributed mainly in the East Mediterranean to central Asia and Mongolia [1, 2]. The name *Hyssopus* is derived from Hebrew ezob which means “sacred herb.”

*Hyssopus* is linked to several folk medicinal uses, providing several biologically active constituents especially the main compounds from essential oils. The essential oil is primarily consumed for flavoring, preservation of food, and other therapeutic uses [3].

Essential oils from the plants are generally recognized as safe for consumption if consumed in limited or recommended doses. Hence, the safety profile of the hyssop extracts is important in various diseases. *Hyssopus* spp. possessed curative properties against cough, cold, loss of appetite, fungal infection, and spasmodic condition. Essential oil constituents also exhibited antimicrobial, antifungal, and muscle relaxant properties [4]. To the best of our knowledge, there is no literature review considering the essential oils and their health-promoting effects and safety profile of *Hyssopus* genus.

This comprehensive review provides insights into the chemical composition, method of extraction, pharmacological properties, adverse effects, and toxicological data of essential oils extracted from *Hyssopus officinalis*.

## 2. Review Methodology

Scientific search engines PubMed and ScienceDirect were searched to retrieve literature and cross-references using the next MeSH keywords: “Lamiaceae”, “Lamiaceae/chemistry”, “Hyssopus Plant/chemistry”, “Oils, Volatile/analysis”, “Limonene”, “beta-pinene”, “pinocamphone”, “Monoterpenes”, “Oils, Volatile/pharmacology”, “Anti-Infective Agents/isolation & purification”, “Anti-Infective Agents/pharmacology”, “Antioxidants/chemistry”, “Antioxidants/isolation & purification”, “Antioxidants/pharmacology”, “Acetylcholine/pharmacology”, “Animals”, “Muscle Relaxation/drug effects”, “Apoptosis/drug effects”, “Cell Lines”, “Antitumor”, “Oils, Volatile/adverse effects”, “Oils, Volatile/toxicity”, and “Seizures/chemically induced”.

For this comprehensive review, extenso papers written in the English language were and abstracts, communications, and research with homoeopathic preparations were excluded. The taxonomy of plants has been validated using The Plant List database (http://www.theplantlist.org/) [5, 6].

## 3. Botanical Description and Distribution

Among the medicinal and aromatic plants, *Hyssopus* spp. have not been studied extensively. The known accepted species and subspecies are *H. ambiguus*, *H. cuspidatus*, *H. latilabiatus*, *H. macranthus*, *H. officinalis*, *H. officinalis* subsp. *aristatus* (Godr.) Nyman, *H. officinalis* subsp. canescens (DC.) Nyman, *H. officinalis* subsp. montanus (Jord. & Fourr.) Briq., *H. seravschanicus*, and *H. subulifolius* (Rech.f.) Rech.f. [7–11].

The genus *Hyssopus* had six species that were used as herbal remedy like *H. officinalis* which is utilized as a condiment in a food factory and as a flavoring agent [12]. The other five species were *Hyssopus ambiguus* (Trautv.) Iljin ex Prochorov. & Lebel, *Hyssopus cuspidatus* Boriss, *Hyssopus latilabiatus* C.Y.Wu & H.W.Li, *Hyssopus macranthus* Boriss., and *Hyssopus seravschanicus* (Dubj.) Pazij.

The geographical distribution of *Hyssopus* spp. is shown in Table 1.

*Hyssopus officinalis* is widespread in the Mediterranean countries [14] and central Asia and north-west India [15]. Traditionally, hyssop seedlings are transplanted into the field with the first harvest occurring two years later, immediately after bloom [16]. Several subspecies have been recorded especially for Europe and northern Africa [17]. Nevertheless, several authors do not formally recognize any intraspecific ranks within this taxon, stating that variations displayed by the species are linked to local ecological conditions [18].

The flowering tops and leaves are used as flavors in food and beverages and cosmetic product preparations [19]. Flowers are useful in attracting bees for pollination and make them an important ornamental plant. Several types, differing in flower color, flowering time, and leaf shape are available commercially: “alba” (white flowers), “grandiflora” (large flowers), “rosea” (rose flowers), and “rubia” (red flowers) [16].

The essential oil from the herb is commonly used in cosmetics, perfumes, alcoholic and nonalcoholic beverages, and food additives. Dry leaves of hyssop are consumed as spices and herbal tea [14]. The seeds are often mixed, and it is quite difficult to obtain uniform plant populations for specific requirements, such as decorative flower production, honey bee forage production, high volatile oil yield, and uniform quantitative oil composition.

The reproduction is made by dividing clumps in late spring, towards April-May, or at the beginning of autumn. Seeds may be sown in March-April in calcareous light soil.

The essential oil productivity ranges between 10 and 20 kg/ha [20]. The productivity of essential oil varies greatly depending on the variation in plant species. In fact, comparing 13 European sources of hyssop, the yield of fresh leaves ranged from 5 to 32 tons/ha, and that of dry leaves from 0.67 to 3.26 tons/ha [19].

The inflorescence is 20-25 cm long, false spikelike, composed of 4 to 10 flowered pseudoverticils in the terminal [21]. Due to the high content of essential oils (0.3–1.0%), one of the most important species among *Hyssopus* is *Hyssopus officinalis* L. H. (hyssop), frequently used as a drug in the pharma industry and as a functional ingredient in the food industry [1, 22].

The root of *H. officinalis* is densely branched with a multiheaded tap root. The stems are 0.5–0.7 m in height, erect, or decumbent, dividing into many woody stems.

The leaves are opposite, shiny dark-green, entire-edged, and lanceolate or oblong, obtuse to acuminate that are 2–4 cm long and 0.5–1 cm wide.

The morphological and genetic hyssop complexity describes its high variability and the existence of numerous geographically different subspecies [23, 24]. *H. officinalis* is largely cultivated but also found in the wild conditions, characterized by a high heterogeneity. The high intraspecific diversity of hyssop is due to morphological differences (leaf, inflorescence, colour of flower, position, and form) and, especially, to biochemical traits (essential oils).

## 4. Phytochemistry and Bioactive Principles

Hyssop oil from different phenotypes or different areas shows great variability in chemical composition [25–28]. The chemical composition can be related to many factors, such as the origin, geography, climate and technological influence, stages of development, parts used, and harvesting time, as well as the presence of chemotypes [29].

The characteristics of genus *Hyssopus* essential oil were analyzed by gas chromatography-mass spectrometry (GC/MS) and GC analysis, and 21 compounds were identified, comprising 95.6% of oil. The compounds included six sesquiterpenes (60.5%), one phenol (0.2%), seven monoterpene hydrocarbons (32.3%), and five oxygenated monoterpenes (60.5%) [30]. Pinocamphone (49.1%), isopinocamphone (9.7%), and *β*-pinene (18.4%) were the major monoterpene constituents [30, 31]. Joulain [32] for the first time identified the constituents of genus *Hyssopus* that were *cis*-pinonic, hydroxyisopinocamphone, pinic acid, *cis*-pinic acid, myrtenic acid, methyl myrtenate, and myrtenol methyl ether. The essential oil's major components were *β*-pinene (10.5 and 10.8%), pinocamphone (34 and 18.5%), camphor (0.3 and 5.3%), linalool (0.2 and 7.9%), and isopinocamphone (3.2 and 29%).

GC and GC-MS analysis of *H. officinalis* volatile oil from Spain showed 1,8-cineole (52.89%) and *β*-pinene (16.82%) [26]. Özer et al. [9] performed GC analysis of *H. officinalis* from Turkey, with 34 components found, and they were 91% of the total detected components. The major components were isopinocamphone (5.3%), pinocamphone (19.6%), pinocarvone (36.3%), *β*-pinene (10.6%), and 1,8-cineole (7.2%) [9, 33]. *Hyssopus officinalis* from the Alps in Banon, France, was analyzed for its essential oil components, its major components were *α*-pinene (2.4%), *β*-pinene (3.0%), limonene (5.4%), linalool (49.6%), 1,8-cineole (13.3%), and *β*-caryophyllene (2.8%), and minor components were pinocamphone and isopinocamphone [34].

The Romanian's *H. officinalis* oil contains mainly aliphatic fatty acids like eicosadienoic acid (0.68%), linolenic acid (63.98%), arachidic acid (2.64%), stearic acid (10.73%), and palmitic acid (15.60%) [35]. But the plants of genus *Hyssopus* of Iran possessed different constituents and their concentration was found as bornyl acetate (1.42%), *β*-caryophyllene (2.10%), *β*-bourbonene (1.47%), camphor (6.76%), caryophyllene oxide (2.13%), spathulenol (2.14%), germacrene (3.39%), and myrtenyl acetate (74.08%) [36].

Chemical analysis of the *H. officinalis* endemic in former Yugoslavia shows that the essential oils are composed mainly of *cis-* and *trans*-pinocamphone and pinocarvone, together with lesser amounts of germacrene D, bicyclogermacrene, elemol, and spathulenol [11].

Essential oils isolated from plants of hyssop grown in two different localities near Urbino (Italy) that showed major components were pinocamphone (34% and 18.5%), isopinocamphone (3.2% and 29%), and *β*-pinene (10.5% and 10.8%). The major differences in the composition of the oils were detectable in the ratio of pinocamphone/isopinocamphone, in the percentage of linalool (0.2% and 7.9%) and camphor (0.3% and 5.3%) [11].

Oil from hyssop found in Poland contained 52 compounds, including 1 unidentified. The main components of hyssop essential oil were isopinocamphone (22.53–28.74%), pinocamphone (11.41–17.99%), *β*-pinene (6.69–12.01%), elemol (5.02–7.57%), germacrene D (3.14–6.98%), and bicyclogermacrene (2.55–4.36%) [37]. Garg et al. [30] found that the main volatile constituents of *H. officinalis* essential oil from a variety of locations included *β*-pinene, limonene, *β*-phellandrene, 1,8-cineole, pinocamphone, isopinocamphone, pinocarvone, germacrene-D, and methyleugenol. They also found that the oils extracted from different subspecies or plant populations of varying origin or morphology differed in percentage composition of the major volatile constituents.

Said-Al Ahl et al. [38] from Egypt found that the major constituents of *H. officinalis* oil were *cis*-pinocamphone (26.85%), *β*-pinene (20.43%), *trans*-pinocamphone (15.97%), *α*-elemol (7.96%), durenol (3.11%), *β*-phellandrene (2.41%), caryophyllene (2.34%), (E)-2,6-dimethyl-1,3,5,7-octatetraene (2.27%), 3(10)-caren-4-ol, acetoacetic acid ester (2.14%), bicyclogermacrene (1.83%), myrtenol (1.73%), germacrene D (1.68%), limonene (1.56%), *γ*-eudesmol (1.36%), and linalool (1.08%).

Ahmadi et al. [39] conducted a study on improving the yield of essential oil from *H. officinalis* after first and second harvest. It was concluded that citrulline application at the rate of 2 mM resulted in 15 and 30% of improvement in the yield after the first and second harvest, respectively. The authors found that the application of 2 mM of citrulline yielded 47% isopinocamphone as the dominant essential oil component under severe drought from *H. officinalis*. The principal components present in *Hyssopus* essential oil are presented in Table 2.

**Figure 1:**
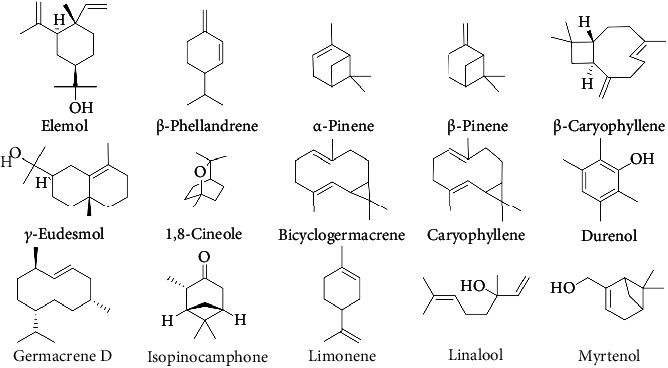
Chemical structures of the main bioactive constituents of *Hyssopus* essential oil.

The main constituents of *Hyssopus* essential oils and their chemical structures are shown in Figure 1.

## 5. Extraction Methods of Essential Oil from *Hyssopus* Genus

*Hyssopus* essential oils are usually extracted using traditional method such as Soxhlet extraction and hydrodistillation whereas nonconventional methods such as supercritical fluid extraction (SFE), ultrasound-assisted extraction (UAE), and instant controlled pressure drop process (DIC) have been utilized recently to improve the yield of essential oils from *Hyssopus* aerial plant parts [23].

In a recent study, the essential oil components were extracted using supercritical carbon dioxide (SC-CO_2_) extraction and Soxhlet extraction from 4 species of genus *Hyssopus* (differentiated by the corolla color). The extraction showed that the major components of genus *Hyssopus* species were pinocarvone, pinocamphone, and isopinocamphone. SC-CO_2_ results showed a lesser presence of monoterpene hydrocarbons and a high amount of oxygenated hydrocarbons [44].

In a recent study, supercritical fluid extraction (SFE) was used for the extraction of essential oil components from hyssop and yielded pinocamphone in the concentration of 0.7 to 13.6%, sabinene (4.2 to 17.1%), and *iso*-pinocamphone (0.9 to 16.5%). For the highest extraction of isopinocamphone and pinocamphone, the optimized conditions were 45°C temperature; 100 atmospheric pressure, with methanol 4.5 *μ*L (0.14% *w*/*w*); 25 min static extraction time; and 20 min dynamic extraction time [45]. The authors concluded that input factors such as pressure, temperature, static and dynamic time, and modifier affect the extraction of essential oil components from *Hyssopus*.

Kazazi and Rezaei [46] collected hyssop from Iran which after analysis showed the presence of a high concentration of sabinene (11.04%). The major compounds of both extractions SFE and Soxhlet extraction in comparison were *β*-pinene (1 to 6%), terpinene-4-ol (4 to 10%), and 1, 8-cineol (eucalyptol) (60 to 75%) [47, 48]. The extraction methods, agrotechnical factors, climatic parameters, and from one growing season to another were the factors that vary the essential oil content of extract [27, 49–51].

In another study, essential oil from *H. officinalis* was extracted using an instant controlled pressure drop process (DIC). GC-MS analysis showed that 65% of total constituents of chromatogram were *cis*-pinocamphone (21.59%), *trans*-pinocamphone (7.93%), elemol (7.12%), bicyclogermacrene (6.58%), germacrene D (6.52%), limonene (6.36%), *β*-pinene (5.20%), and *trans*-caryophyllene (4.65%). Essential oil components such as linalool, 3-octanone, *α*-pinene, and *α*-thujene were detected in abundance in DIC compared to the hydrodistillation and ultrasound-assisted extraction.

## 6. Biological Activities

The essential oil and the extracts isolated from plants demonstrated average *in vitro* antibacterial activity, antimycotic insect killing, and antiviral properties [52, 53]. Hyssop essential oil extracts showed excellent antioxidant and anticancer activities [28, 54], although there is significant potential that has yet to be investigated [55, 56].

### 6.1. Antimicrobial, Antifungal, and Antiviral

The essential oil of *Hyssopus* contains *β*-pinene, iso pinocamphone, terpinene-4-ol, carvacrol, pinocarvone, pinocamphone, and *p*-cymene compounds with antimicrobial properties, which could be used to new drug development for therapy or prophylaxis against human infectious diseases [57]. In a recent study, hyssop essential oil demonstrated activity against pathogenic microbes against *Staphylococcus aureus*, *Escherichia coli*, *Bacillus cereus*, *Proteus hauseri*, *Listeria monocytogenes*, *Rhodococcus equi*, *Listeria ivanovii*, *Salmonella enteritidis*, *Enterococcus faecalis*, *Listeria innocua*, and *Bacillus spizizenii* [58].

*Hyssopus officinalis* essential oil holds antifungal activity which is particularly efficient in the reduction of the expansion of fungal species such as *Candida krusei*, *Candida albicans*, and *Candida tropicalis* [10, 58]. The MIC of *H. officinalis* essential oil in a liquid medium for yeast was between 0.15 and 0.3%. The essential oil also exhibited antimycotic activity against 10 yeast and fungi and *C. albicans* in the range of 15.625 to 250 *μ*L/mL [59]. In Serbia, the mushroom *Agaricus bisporus* (Lange) Imbach was destroyed by the fungus *Mycogone perniciosa* (Mang); in 2005, the research was conducted to find out the antifungal agent to inhibit its growth.

Then, the essential oil of *H. officinalis* was tested for its antiperniciosa activity; the results showed minimal fungicidal activity of about 15 to 20 *μ*L/mL and MIC about 5 *μ*L/mL [60]. Then, aromatic plants were tested for their antifungal activity, to enhance the growth of mushroom [60, 61]. Then, from the aromatic plants of Mediterranean area, 12 essential oils were tested against four fungi, which were *Penicillium Italicum*, *Botrytis cinerea*, *Rhizopus stolonifer*, and *Phytophthora citrophthora*. The conclusion revealed that the hyssop oil exhibited weak to moderate fungicide activity but they could act as a food product preservative [4]. By using the agar diffusion method, *H. officinalis* essential oil showed complete growth inhibition of *Aspergillus niger* in concentrations between 0.5 and 1.5% [62].

*Hyssopus officinalis* dried leaf extract also showed efficacy in the replication inhibition of HIV (human immunodeficiency virus) [63].

### 6.2. Antioxidant

2,2-Diphenyl-1-picrylhydrazyl (DPPH) and 2,2′-azino-bis(3-ethylbenzothiazoline-6-sulfonic acid (ABTS) assays are generally used for measuring the antioxidant activity of the plant extracts. Hyssop essential oil reported lower antioxidant activity against DPPH (1.21 *μ*mol Trolox equivalent (TE)/g) and reported approximately ten times higher scavenging activity towards ABTS (10.6 *μ*mol TE/g) [15].

In another study, the hyssop extracts from Serbia and Turkey showed an IC_50_ value of 156.60 mg/mL and 16.37 mg/mL [8, 57]. The differences in the antioxidant properties are due to genetic factors, environmental conditions, and geographic origin [28]. Antioxidant activity of the hyssop essential oil attributed towards the chelating power of transition metals. The antioxidative properties of hyssop essential oil can find important application in preservation of the food items such as ground beef by inhibiting the growth of pathogenic bacteria [64].

### 6.3. Anticancer/Cytotoxic

Natural products are a rich source of compounds with many applications in cancer chemotherapy [65–67]. Moreover, the broad spectrum of natural compounds provides important compounds for therapeutic refinement through molecular modifications [68, 69].

Hyssop extracts were evaluated for anticancer activity against human cancer cell lines using 3-(4,5-dimethylthiazol-2-yl)-2,5-diphenyl tetrazolium bromide (MTT) assay [15]. Authors utilized human breast cancer (MDA-MB 231), malignant melanoma (A375), and human colon cancer (HCT116 lines) cell lines for their investigation. The essential oil extracts from hyssop showed a concentration-dependent inhibitory effect against cancer cell lines. They reported IC_50_ values of 62.66, 35.16, and 29.91 *μ*g/mL MDS-MB 231, A375, and HCT116 cell lines, respectively. High antioxidant activity was suggested to be due to the components of hyssop essential oil such as linalool and methyl eugenol. In another study, *Hyssopus* essential oils showed antitumor activity against tumor cells lines (HeLa, MRC-5, and MDA-MB231) [28]. On the same way, *H. officinalis* subsp. *aristatus* shows slight cytotoxicity towards the A549 cell line (adenocarcinomic human alveolar basal epithelial cells) [70].

The cytotoxicity of marrubiin was studied in 66 cancer cell lines without showing toxic activity; however, *in vivo*, an LD_50_ of 370 mg/kg body weight for marrubiin had been documented [71]. Recent studies have shown a safety limit of up to 100 mg/kg body weight when injected into mice [72].

### 6.4. Mosquito Larvicidal Activity

Mosquitoes are one of the emerging causes of death in developing and underdeveloped countries. Control of mosquito vectors is a challenging task due to environmental concerns and the development of pesticide resistance. Hence, researchers are in search of new botanicals for the control of mosquito-related diseases such as malaria, chikungunya, Zika virus, and dengue [73].

Essential oil of *H. officinalis* demonstrated lethal concentration (LC_50_) values more than 90 *μ*L/L in an acute toxicity research study against double blends of *Culex quinquefasciatus* agent, a vector of lymphatic filariasis, maintaining it as environment-friendly, efficient, and inexpensive mosquito larva killer [74]. The study is crucial for the development of newer and safer mosquitocidal biopesticides for the control of mosquito-borne diseases.

### 6.5. Miorelaxation Activity

An experiment was carried out for testing the sedative and simulative effect of essential oil by inhaling in mouse forced swimming test [75]. The hyssop essential oil inhalation increased the sessile state, and this condition was artificially overagitated by caffeine parenteral injection. The result showed that hyssop essential oil could produce a sedative effect [75].

Lu et al. [76] designed an experiment for evaluation of the myorelaxant effect of *H. officinalis* essential oil on the intestinal muscle sample of rabbit and guinea pigs. The myorelaxing effect of essential oil was due to its component isopinocamphone. BaCl_2_ was involved in myocontraction in the ileum (IC_50_ = 48.3 and 70.4 *μ*g/mL), and isopinocamphone did myorelaxation by the inhibition of acetylcholine on ileum muscles (IC_50_ 42.4 and 61.9 *μ*g/mL).

*H. officinalis* oil contains limonene and *β*-pinene which produced no effect on muscle contraction, but the synergic effect of components could not be excluded. The researchers believed that hyssop oil could induce myorelaxation after the alteration of ion channels and interaction with plasma membrane. The synergic activity of components of essential oil depended upon chemical structure, lipophilic nature, and subcomponents of essential oil such as interaction of *β*-pinene and limonene [76].

A recent study of extract of *H. officinalis* on seizures induced by pentylenetetrazole and hippocampus mRNA level of iNOS in rats found that 100 mg/kg dose of hyssop extract might have anticonvulsant effects. However, these anticonvulsant effects might not occur through the iNOS gene expression [77].

**Figure 2:**
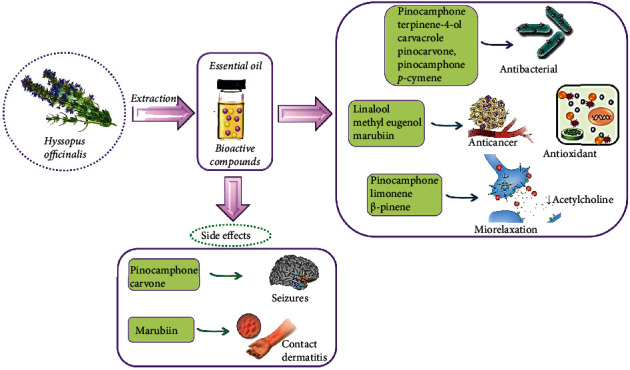
Schematic representation of biological actions, side effects, and the correlation with the most representative bioactive compounds isolated from *Hyssopus officinalis* essential oil. Biological activities of *Hyssopus* essential oils

The biological activities of *Hyssopus* spp. essential oils are summarized in Figure 2.

## 7. Safety Data and Adverse Effects

A survey of the literature documented essential oils of hyssop and other 10 plants: eucalyptus, fennel, hyssop, pennyroyal, rosemary, sage, savin, tansy, thuja, turpentine, and wormwood, to be powerful convulsants due to their content of highly reactive monoterpene ketones, such as camphor, pinocamphone, thujone, cineole, pulegone, sabinyl acetate, and fenchone. The hyssop essential oil contains one or more oxygenated monoterpenes, such as pinocamphone.

The epileptogenic properties of these convulsants have been well established and studied in animals [78]. They may interact with and exert pharmacological action on central nervous system targets involved in epilepsy [79, 80]. It was shown by electrocorticographic records that Wistar rats, after injection of 80 mg/kg hyssop essential oil, develop subclinical generalized sustained high-voltage spikes. Increasing the dose to 1.25 g/kg leads to rhythmic myoclonus, subsequently to one or several tonics, clonic, ortonic-clonic seizures, and eventually lethal convulsive status. Similar results were obtained with half these doses of pinocamphone that produced seizures when used both internally and topically [81]. Also, a carvone derivative, hydroxy dihydrocarvone that increases seizure latency at high doses, may come with negative side effects, including palpebral ptosis, decreased response to touch, and decreased motor activity [82].

Hyssop essential oil modulates structures involved in neurotransmitter release and metabolism such as N-methyl-D-aspartate receptor (NMDA), gamma-aminobutyric acid type A (GABAA) and type B (GABAB), glycine, and acetylcholine receptors, as well as the acetylcholine esterase and GABA transaminase enzymes. Hyssop essential oils also modulate voltage-gated sodium and calcium channels and affect neuronal excitability and action potential dynamics. Another key property is the inability to cross the blood-brain barrier (BBB) of hyssop essential oil [79].

Other possible side effects of hyssop include allergic reactions. It can irritate the respiratory tract due to vexation of mucous and bronchospasm by marrubiin—another component of hyssop essential oil.

Hyssop oils are often used in medical aromatherapy, like other plant oils from the Lamiaceae: basil, lavender, peppermint, rosemary, or thyme. However, the side effects of aromatherapy have been suppressed or refuted by many inexpert aromatherapists to date. According to Yoo [83], the most common complication among 3000 aromatherapy patients recorded was a cutaneous adverse reaction, occurring in 0.99% of all participants. Most of these cases are deemed contact dermatitis caused by an essential oil. The second most common complication was asthmatic symptoms, early menstruation, arrhythmia, and oliguria.

No clinical trials have been conducted on hyssop essential oil adverse effects in humans. Reports are only from clinical research data obtained from hospitals [79]. The metabolites of principal active ingredients of hyssop: *cis*- and *trans*-3-pinanones, were examined from mouse and human liver microsomes by Höld et al. [33]. The major metabolites of *cis*-3-pinanone were 2-hydroxy-*cis*-3-pinanone and two minor metabolites. These compounds exhibited reduced toxicity but also acted as GABAA receptor antagonists. The urine from oral *cis*-3-pinanone treatment contained conjugates of metabolites and 2,10-dehydro-3-pinanone. *Trans*-3-pinanone has slower metabolization than *cis*-isomer and produces different hydroxy derivatives.

## 8. Limitations

In general, at the right dose, hyssop does not have adverse effects and toxicity. However, most of the herbs are safe when used in moderation, but excessive consumption can produce unknown effects [84]. For this fact, hyssop is no exception. This herb is believed to be relatively safe at levels commonly used in foods. The appropriate dose of hyssop depends on several factors such as the user's age, health, and several other conditions. At this time, there is not enough scientific information to determine an appropriate range of doses for this plant. However, at high doses, hyssop oil may be dangerous due to various other side effects, mainly drowsiness, seizures, and sensory effects associated with psychological disorders.

Although herbal products may offer a benefit, it is important to detect even small risks that would significantly affect the risk-benefit ratio in pregnancy. There is very little research on how hyssop products may affect pregnancy (it might cause the uterus to contract or start menstruation).

Although hyssop products may offer a benefit, it is important to detect even small risks that would significantly affect the risk. In addition, herb interactions with conventional medications may provoke undesirable effects. Several interactions have been described between herbal drugs and antidepressants or psycholeptics [85].

Despite the promising medical effects achieved in various researches, yet further detailed studies are required to better clarify the side effect of hyssop. The use of herbal medications does not have strict regulations. This problem is still poorly recognized and underestimated [78]. Since herbal medicines are a part of traditional medicine, they are not included in the FDA or EFSA categories giving a false impression of safety.

Therefore, in general, there is a need for the regulation of hyssop drugs to ensure their safety and determine the efficacy and constituents of the preparations [84]. Physicians should consider balancing the risks and benefits of complementary and alternative medicine using hyssop and its active compounds [86].

## 9. Overall Conclusions

Hyssopus essential oils are usually extracted using conventional methods such as hydrodistillation. However, recently, nonconventional methods such as SFE, UAE, and DIC are getting popularized due to higher extraction efficiencies compared to conventional methods. Most of the recent and older studies used GC and GC-MS techniques for qualitative and quantitative characterization of the essential oil from the *Hyssopus* genus. The genus *Hyssopus* had essential constituents which perform multiple actions. Its essential oil constituents possessed curative properties against cough, loss of appetite, fungal infection, spasmodic condition, and potent antimicrobial activities. The other *in vivo* actions are the sedative effects, plasma membrane relaxation, and cytotoxic activity. Biological activities and aroma from essential oil suggest its use as a potential antioxidant food ingredient and other pharmaceutical drugs.

At the same time, well-designed clinical trials examining the described characteristics of hyssop extracts and possible adverse reactions are still not investigated and need more studies to complete whether biological differences in results of the researches reverse the various types of plant material used, different procedures of isolation, different chemotypes, collection time, and locations. Further, molecular insights into various biological activities of essential oil from *Hyssopus* genus need to be explored in more depth.

## Figures and Tables

**Table 1:** Species and distribution area of *Hyssopus* spp. [13].

Species	Distribution area
*Hyssopus ambiguus* (Trautv.) Iljin ex Prochorov. & Lebel	Altai Republic, Kazakhstan
*Hyssopus cuspidatus* Boriss.	Altai Republic, Kazakhstan, Xinjiang, Mongolia
*Hyssopus latilabiatus* C.Y.Wu & http://h.w.li/	Xinjiang
*Hyssopus macranthus* Boriss.	Altai Republic, Siberia, Kazakhstan
*Hyssopus officinalis* L.	Europe, Algeria, Morocco, Iran
*Hyssopus seravschanicus* (Dub.) Pazij	Afghanistan, Pakistan, Kyrgyzstan, Tajikistan

**Table 2:** Principal bioactive compounds of *Hyssopus* essential oil.

Plant material	Essential oil yield	Principal component of essential oil	Region	Reference
Air-dried aerial parts	1.2%	*Cis*-pinocamphone (21.59%), *trans*-pinocamphone (7.93%), elemol (7.12%), bicyclogermacrene (6.58%), germacrene D (6.52%), limonene (6.36%), *β*-pinene (5.20%), *trans*-caryophyllene (4.65%)	Iran	[40]
Aerial parts	0.18%	Myrtenyl acetate (74.08%), camphor (6.76%), germacrene (3.39%), spathulenol (2.14%), caryophyllene oxide (2.13%), *β*-caryophyllene (2.10%), *cis*- sabinol (1.75%), *β*-bourbonene (1.47%), bornyl acetate (1.42%)	Iran	[39]
Fresh aerial part (stem with inflorescence)	0.47%	Pinocamphone 19.8%, *β* − pinene ≤ 20.5%, *iso*-pinocamphone 24.6%	Spain	[3, 41]
Aerial part (blue flowers)	0.36%	Hedycaryol ≤ 9.1%, germacrene D ≤ 5.5%, *β* − pinene ≤ 11.4%, isopinocamphone ≤ 33.6%, pinocarvone ≤ 28.1%	East Lithuania	[41, 42]
Aerial parts	0.4%	Limonene ≤ 9.8%, germacrene D ≤ 11.3%, 1, 8 − cineole ≤ 11.9%, pinocamphone ≤ 64.9%, *β* − pinene ≤ 32.4%	India	[41, 43]
